# An approach for assisting diagnosis of Alzheimer's disease based on natural language processing

**DOI:** 10.3389/fnagi.2023.1281726

**Published:** 2023-11-16

**Authors:** Ning Liu, Lingxing Wang

**Affiliations:** ^1^School of Science/School of Big Data Science, Zhejiang University of Science and Technology, Zhejiang, China; ^2^Department of Neurology, Second Affiliated Hospital of Fujian Medical University, Quanzhou, Fujian, China

**Keywords:** Alzheimer's disease, natural language processing, linguistic features, deep learning, LSTM

## Abstract

**Introduction:**

Alzheimer's Disease (AD) is a common dementia which affects linguistic function, memory, cognitive and visual spatial ability of the patients. Language is proved to have the relationship with AD, so the time that AD can be diagnosed in a doctor's office is coming.

**Methods:**

In this study, the Pitt datasets are used to detect AD which is balanced in gender and age. First bidirectional Encoder Representation from Transformers (Bert) pretrained model is used to acquire the word vector. Then two channels are constructed in the feature extraction layer, which is, convolutional neural networks (CNN) and long and short time memory (LSTM) model to extract local features and global features respectively. The local features and global features are concatenated to generate feature vectors containing rich semantics, which are sent to softmax classifier for classification.

**Results:**

Finally, we obtain a best accuracy of 89.3% which is comparative compared to other studies. In the meanwhile, we do the comparative experiments with TextCNN and LSTM model respectively, the combined model manifests best and TextCNN takes the second place.

**Discussion:**

The performance illustrates the feasibility to predict AD effectively by using acoustic and linguistic datasets.

## 1 Introduction

As we know, Alzheimer's disease (AD) is a chronic and progressive disease, and the group of people with AD is expanding with the increase in the aging population. Longfei et al. (2020) reported that there were 15.07 million people over 60 years old suffering from dementia in China in 2020, including 9.83 million AD patients and 3.92 million vascular dementia. Meanwhile, there are over half a billion AD patients in America nowadays^1^. AD has become a worldwide problem, and it is estimated that there will be approximately 7.6 billion patients falling ill with AD or other dementias by 2030. Although clinicians can differentiate AD people from healthy controls by a combination of cognitive test scales (Mesulam et al., 2012), it is usually time-consuming and inaccurate. Therefore, it is essential to develop a more reliable but simple method to differentiate different cognitive impairments, especially for the early diagnosis of AD.

A precious study in this area mainly includes two methods. The first one is feature extraction manually combined with machine learning to recognize AD properly, and it usually needs expertise and knowledge in order to extract more distinguishing features, so the accuracy and integrity of features cannot be guaranteed. The second one is deep learning, which uses a powerful deep learning model with multiple hidden layers without feature engineering, and a deep neural network can learn feature representations from datasets by using cascades of multilevel non-linear processing units for feature extraction. Moreover, the performance of deep learning is usually better than the first method, but the interpretability of the second method is poor.

The first method includes the conventional linguistic features extraction manually and the machine learning method. For example, Fraser et al. (2016) extracted mel-frequency cepstral coefficient (MFCC) features (Chen et al., 2014) on Pitt datasets, and this method was the first to carry out acoustic-prosodic analysis to recognize AD patients. Roark et al. (2011) employed natural language processing (NLP) and automatic speech recognition (ASR) to differentiate mild cognitive impairment (MCI) from healthy controls (HC), and the extracted features included pause frequency and duration. Finally, they used the SVM classifier combining the extracted features and yielded the best area under curve (AUC) of 0.861 by combining automated speech, linguistic features, and cognitive test scores. Zehra et al. (2021) extracted graph-based features encoding patterns and speech rate (Luz, 2013) from the Carolina Conversations Collection (Pope and Davis, 2011) and employed a logistic regression classifier to achieve a best accuracy of 85% when distinguishing AD from NC. Guo et al. (2021) extracted low-level acoustic features such as the number of utterances, speech rate, and vocal events, then employed the Bayesian classifier to train on low speech datasets extracted from the recordings, and finally obtained an accuracy of 68% for classifying AD and older adults controls. Antonsson et al. (2020) measured semantic ability quantitatively and employed the support vector machine (SVM) to recognize AD from NC, and the area under curve (AUC) value is 0.93. Clarke et al. (2021) measured 286 linguistic features to train the SVM model, and the final accuracy is 62–78% for AD+MCI vs. HC, 59–90% for AD vs. HC, and 50–78% for MCI vs. HC. Shamila et al. (2021) investigated conversational features such as disfluency, overlap, pause, and other elements in AD detection and employed Carolinas Conversations Collection Classifier (Zehra et al., 2021), and finally achieved an accuracy of 90% on Alzheimer's Dementia Recognition through Spontaneous Speech (ADReSS) datasets. Jarrold et al. (2014) extracted acoustic and linguistic features combined with the logistic regression classifier and achieved the best accuracy of 85.4% on DementiaBank datasets. Haulcy and Glass (2021) extracted features of x-vectors and i-vectors (Snyder et al., 2018) for tackling AD detection and phonetic features and obtained an accuracy of 85.4% in AD detection with SVM and random forest classifier.

In recent years, the deep learning method for AD diagnosis has become popular. For example, Aparna et al. (2021) pretrained the Bidirectional Encoder Representations from Transformers (BERT) model to recognize AD with ADReSS datasets and finally obtained an accuracy of 83.33%, which is better than feature extraction manually. The study (Chen et al., 2019) proposed an attention network composed of GRU and CNN modules and obtained an accuracy of 97% in distinguishing AD from normal controls. Amit et al. (2021) used the fastText and CNN models to recognize AD, respectively, and the fastText model obtained a best of 83.3% accuracy which is better than the performance of acoustic and linguistic features manually. The study (Fritsch et al., 2019) trained the LSTM model with the n-gram language model and obtained the best accuracy of 85.6%. Guo et al. (2021) used the BERT model on ADReSS and DementiaBank datasets and also obtained a competitive result.

The Text Convolutional Neural Network (TextCNN) was first proposed by Kim (2014) in the CV field in 2014, and the model used the convolution windows of different sizes to extract the local semantic information from the text vector and obtained the text representation after the pooling layer and fully connected layer; finally, they made a classification after the Softmax classifier. TextCNN has a better performance in many text classification tasks. For example, Kalchbrenner et al. (2014) proposed a dynamic convolutional neural network (DCNN) model that used dynamic K-max pooling in the pooling layer to obtain better adaptability for sentence modeling. Zhang et al. (2015) proposed a CNN text classification model with the character level, and the model can achieve an ideal classification effect without syntactic and semantic information. Compared with TextCNN, the module is more robust when faced with typos and emoticons. Liu and Guo (2019) extracted features for text sequence by using LSTM and the attention mechanism which could understand the contextual semantics of text. Although TextCNN learned the features of text sequences, it could only capture shallow meanings and was unable to mine deep semantic information. However, as far as we know, TextCNN was not applied to AD diagnosis by transcript.

With the development of science and technology, as well as the enhancement of computing power, large-scale pretrained language models have developed rapidly. In 2018, Devlin et al. (2019) introduced BERT, a self-coding language model that swept the natural language processing (NLP) tasks and redefined several records in this field. The BERT model used the multi-layer encoder of a bidirectional transformer and first proposed the next sentence prediction task and sentence mask innovatively. In this study, we propose a deep learning model that combines TextCNN and LSTM. The extracted features consider both short-term and long-term features. At last, we obtained the best accuracy of 89.3%, which is more competitive than other studies. The performance is better than the TextCNN and LSTM models, respectively, which means the extracted features are more comprehensive than both models. We also list many studies in this area with deep learning and machine learning methods, and the performance of our study is the most competitive. The contributions of this study are as follows:

- To propose a novel deep learning model combining TextCNN with LSTM for the diagnosis of AD.
- To build a model with excellent classification performance in a challenging scenario.
- To compare the proposed model with other machine learning and deep learning models to detect AD using short-term and long-term features obtained from transcripts.

## 2 Materials

### 2.1 Pitt corpus

**Figure 1:**
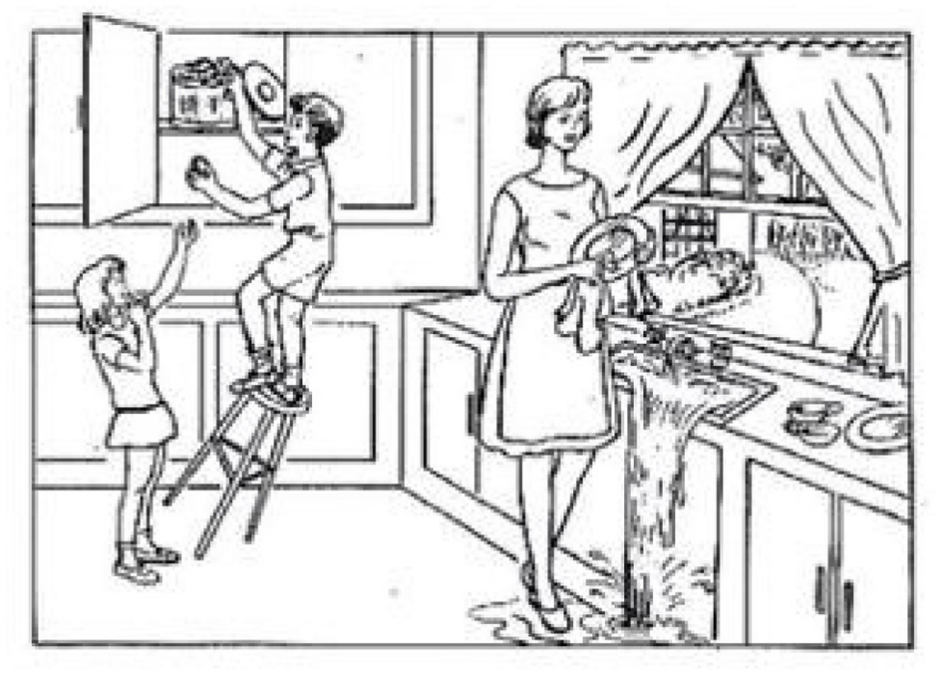
Cookie theft picture.

**Figure 2:**
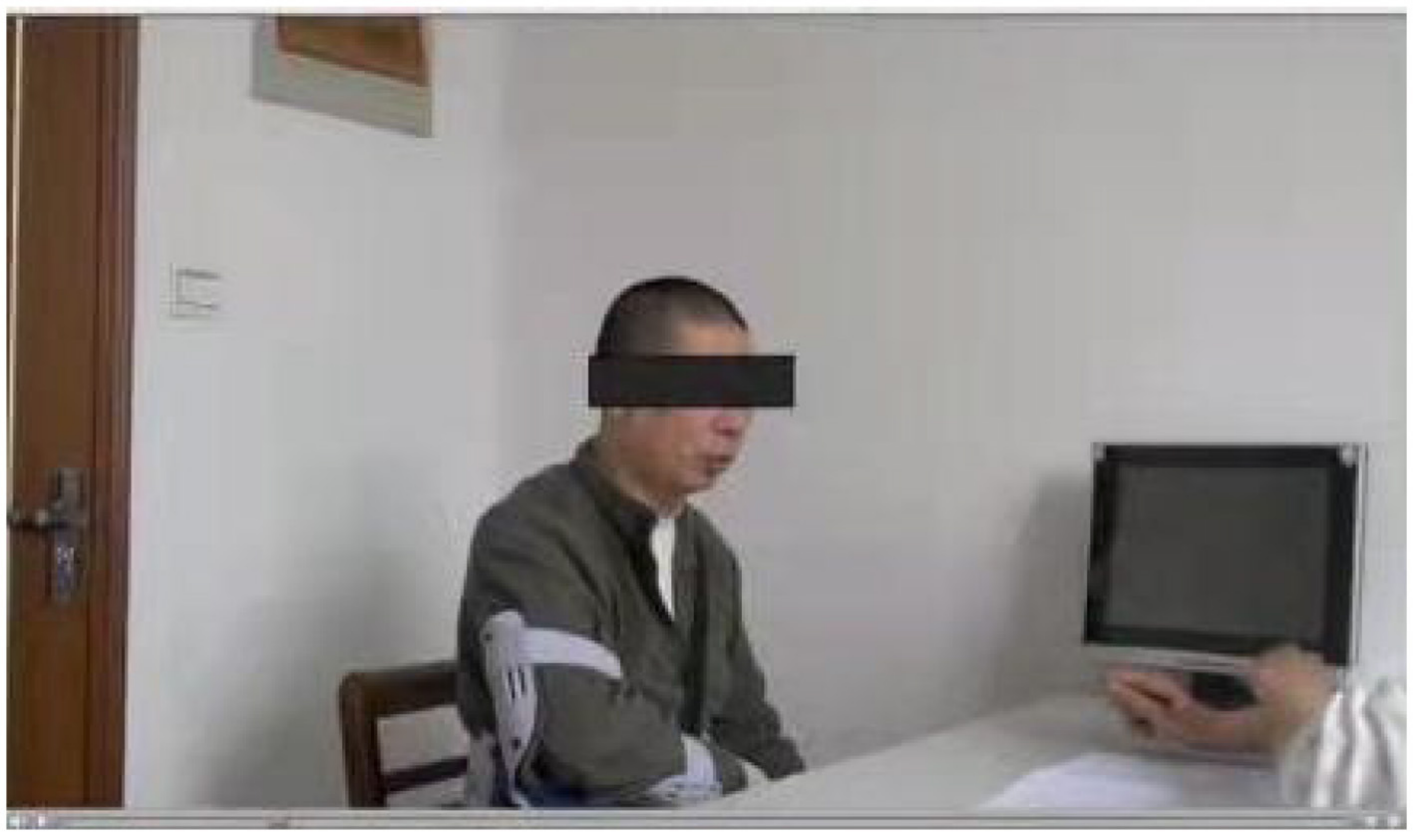
Process of the experiment.

The data used in this study is obtained from the DementiaBank website (https://sla.talkbank.org/TBB/dementia/English/Pitt) (Becker et al., 1994). Figure 1 is a cookie theft picture from the Boston Aphasia Examination (Chen et al., 2019), which was designed by Goodglass and Kaplan in 1972. The experiment is processed in a quiet room, where the participant is shown the picture and asked to describe the picture as detailed as possible (Figure 2), and it is a natural conversation between the doctor and the participant without tips during the process. Then, the corpus is transcripted through the CLAN system (Lin and Chen, 2018), which is a language manage software for transcribing the speech into professional datasets through recording, word segmentation, and parts-of-speech (POS) tagging. The transcripts from voice recordings were gathered from the School of Medicine at the University of Pittsburgh. Every audio file is associated with a transcript; 498 participants in this study were enrolled and obtained the corresponding transcripts after data pre-processing, including 256 people with probable and possible AD and 242 normal controls. The demographic information is shown in Table 1.

**Table 1:** Demographics of Pitt datasets.

	**CTRL (242)**	**Possible/ probable AD (256)**
Age (years)	65.2 (7.8)	71.8 (8.5)
Education (years)	14.1 (2.4)	12.5 (2.9)
Sex (male/female)	86/156	90/166
Mini-mental state exam	29.1 (1.1)	18.5 (5.1)

Tips: CTRL refers to normal controls.

An example of the datasets from Pitt datasets is shown below:

*a little boy is stepping on a ladder that's cockeyed. and the ladder has it's a tripodal it isn't a ladder it's a stool. and it's a a three legged stool. and he's getting cookies outof a jar. and he's handing a cookie to the little girl who's saying “shho” to the mother. and the mother is wiping dishes with water running all over the kitchen floor. and 1pause1. oh god. oh she's she has an open window. and there are bushes in front of the window either in the house next door or some place else. and then there's a tree that doesn't have a trunk.. and. there are two cups and the handles are in opposite directions. and she's sort of dumb because she doesn't turn off the water. she's letting it run on her feet. and half of the kitchen cupboard doors don't have handles. 1pause1. I don't think. and she's wiping the dish with two towels. and she isn't watching her dear darling children fall off the stool. okay isn't that about enough? [sic]*

## 3 Methods

### 3.1 TextCNN

TextCNN is particularly effective in extracting short-term features or local patterns from datasets, and it has become an important feature extractor. The window sliding of TextCNN has no sequence relationship, different convolution kernels do not influence each other; therefore, it has a very high parallel degree. Compared with the traditional Bag-of-Words (BoW) model, TextCNN may be considered more effective in extracting text sequence features (Li et al., 2023). TextCNN includes four layers: convolution layer, pooling layer, fusion layer, and fully connected layer. The model has four filters to obtain multiple features which form the penultimate layer, we extract the linguistic features by convolution operation, the convolution kernel of which only convolution along the time step sequence, the width of every convolution kernel is the same as the dimension of the word vector, mainly for ensuring that the convolution kernel processes the word vector every time, the output is the eigenvector we need. This is the process by which TextCNN extracts features. After the convolution layer, the model extracted the most significant features by the max-pooling layer that can reduce dimension. Finally, a fully connected layer is added to finish the text classification task.

### 3.2 LSTM

A gate mechanism is designed by LSTM to control the flow of information in the network. The gate mechanism consists of specialized neurons that decide which information should be retained or forgotten and where to incorporate the retained information into the next moment. In order to solve the problem of gradient explosion and long-range information disappearance existing in RNN effectively, the LSTM model deals with a long-term dependence based on time series through the operation of three gates, which is crucial for understanding semantics and capturing long-distance features. LSTM is particularly suitable for the linear sequence application scenario of NLP, which makes it so popular in the natural language process (NLP) field.

### 3.3 LSTM-TextCNN

**Figure 3:**
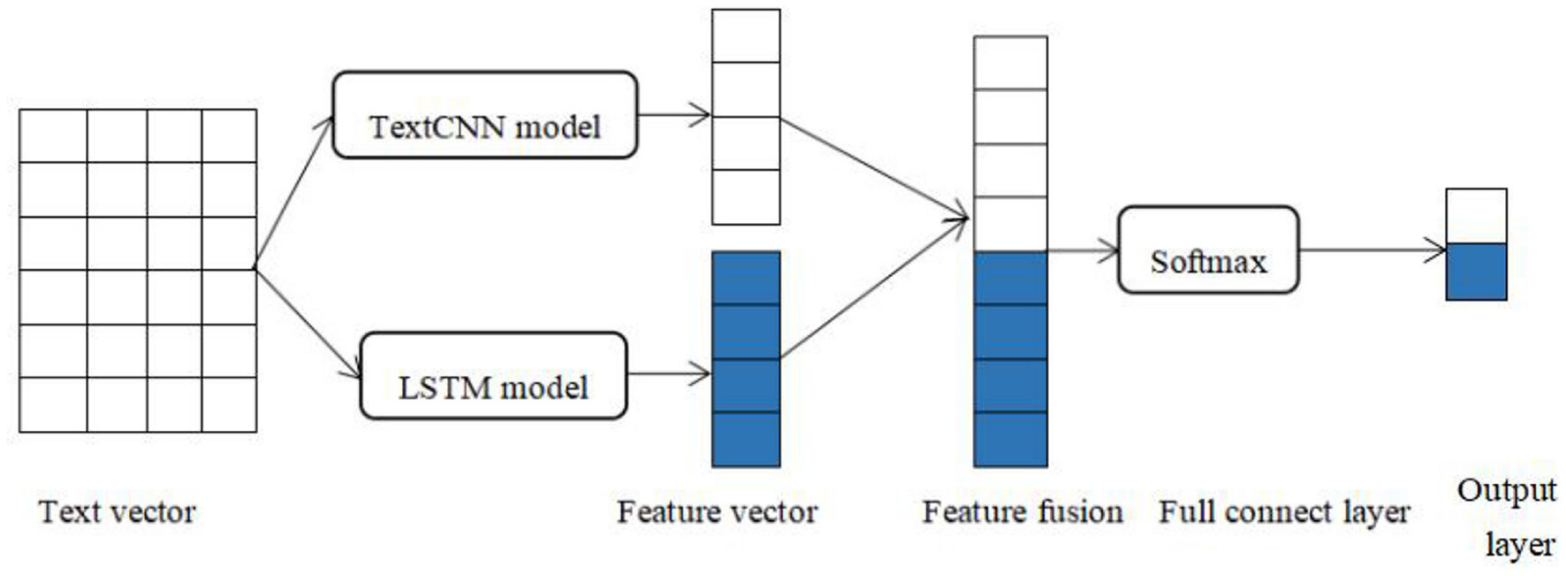
Architecture of LSTM-TextCNN.

LSTM-TextCNN is the combination of LSTM and TextCNN models. The LSTM model can capture long-term dependencies in sentences, establish hidden states in the sequence, and improve the ability of information fusion in the sequence. The TextCNN model can capture local features effectively and calculate in parallel to reduce the computational complexity. The local features extracted from the TextCNN model and the long-distance features from the LSTM model are connected as the input of the next fully connected layer. Specifically, the overall framework of our model is shown in Figure 3.

#### 3.3.1 Input layer

Convert words into vectors using the BERT pre-training model and form a sequential vector as the input.

#### 3.3.2 LSTM layer

Learn information about long-term dependencies and hidden states in sequences.

#### 3.3.3 TextCNN layer

Learn local features and extract key features related to classification.

#### 3.3.4 Model combination

The output vector of the LSTM layer and TextCNN layer is concatenated before the Softmax classifier to get the final feature vectors.

### 3.4 BERT embedding

We fine-tuned the trained BERT base (Devlin et al., 2018), which includes 12-layer, 12-heads, 768-hidden, and 110M parameters. In particular, we used BERT-base uncased, where uncased means that the text has been lower-cased before WordPiece tokenization.

### 3.5 Local features extraction from TextCNN

**Figure 4:**
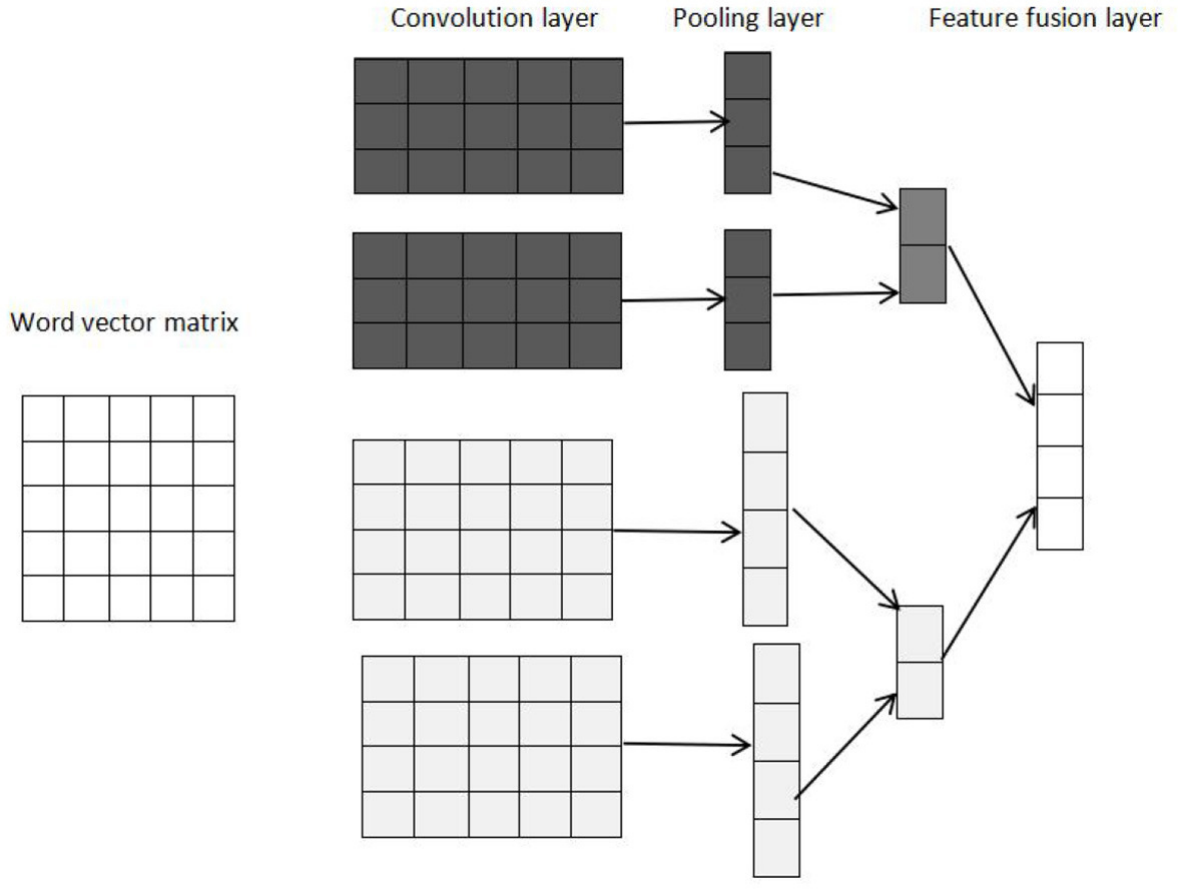
TextCNN model structure.

The local features of the text are extracted by the TextCNN model, the model structure of which is shown in Figure 4. TextCNN is mainly composed of the convolution layer and pooling layer.

In the convolution layer, it first does a convolution operation with convolution kernels of different sizes, and different sizes of convolution kernels represent different receptive fields in order for richer local semantic information. Every convolution kernel scans the input text vector from BERT embedding to obtain the feature vector. In order to improve the expression of the model, the text features extracted by the convolution operation can be transformed by the activation function, and we choose the ReLU function as the activation function because it has a high convergence rate and can solve the problem of gradient disappearance. Then, the TextCNN model further reduces the feature dimension by a max-pooling operation. The pooling layer makes the model pay more attention to the most important features, reduces the feature dimension and the number of parameters, and prevents the occurrence of overfitting to a certain extent. At last, we obtain the final feature vectors by feature fusion layer.

### 3.6 Global features extraction from LSTM

LSTM is a variant of recurrent neural network (RNN) that can learn long periods of dependence. It relies on the structure of some “gates” to allow information to affect the state of each moment selectively and can decide which information should be forgotten or reserved effectively; therefore, LSTM can obtain long distance feature relationships of text sequences. It can remember the past information and also can solve the problem of gradient disappearance in RNN. The global features extracted from the LSTM model, combined with the local features, are used for text classification ultimately, so it is not necessary to collect the output of the model at every moment, but only the output of the last moment of the model, as it includes richer contextual semantic information.

### 3.7 Features fusion module

In the TextCNN model, feature regions with different sizes are extracted by setting different sizes of convolution kernels. However, TextCNN cannot still obtain the dependency between features of different texts due to the particularity of the text, and the features obtained cannot represent the whole text to a certain extent. The memory mechanism of LSTM has great advantages in processing long-term text information, and it can remember past information well. Therefore, we fused both the local and global features in order to obtain the final feature vectors.

The common feature fusion methods include concatenation, bit addition, and average. The original linguistic features can be preserved effectively, and the feature loss is avoided just by concatenating feature vectors simply. Therefore, this study chooses to concatenate the local features extracted from the TextCNN model and global features from the LSTM model into a feature vector. Assume that the feature extracted by the TextCNN model is F_l_, and the feature extracted by the LSTM model is F_g_, and the two features are spliced into a new feature F. Therefore, the feature vector F contains both the local features and the whole features of the text, and then F can be expressed as


(1) $$\begin{array}{l} F=F_{l}⊕F_{g} \end{array}$$

Where ⊕ represents vector concatenate operation.

### 3.8 Classification result

The fused features need to be passed into the fully connected layer first, and the Softmax classifier is used for the classification task. The dimension of the output vector must be the same as the number of categories (2 in this study) in the classification. Finally, we used the feature vector F to do the classification;


(2) $$\begin{array}{l} y=soft\;\max\left(W_{c}F+b_{c}\right) \end{array}$$

where F represents the features after fusion, W_c_ is the parameter, b_c_ is the bias, and y represents the classification result.

### 3.9 Hyperparameter setting

The experiment uses an Intel Core i5 8300H quad-core eight-thread CPU, 4 GB NVIDIA GTX 1050Ti for graphics, Python 3.7 for programming language, and Pytorch 1.7.1+cu101 for deep learning framework. The experiment is completed under the deep learning framework Keras, and we chose the BERT open-source library Pytorch Transformers and the BERT-Base pre-training model, which was released by Google. All the models used in this study are downloaded from the website of Hugging Face (https://huggingface.co/models?pipeline_tag=sentence-similarity&sort=downloads) *via* API (AutoModel.from_pretrained, AutoTokenizere.from_pretrained). The parameters of the training process are shown in Table 2.

**Table 2:** Parameters of the training process.

**Parameters**	**Value**
Epoch	10
Batch size	16
Learning rate	1e-5
Max grad norm	10
Dropout	0.2

Table 3 shows the parameters of the BERT-base used in this study.

**Table 3:** BERT parameters.

**Parameters**	**Value**
vocab_size	30,522
num_attention_heads	12
num_ hidden_layers	12
attention_probs_dropout_prob	0.1
hidden_size	768
intermediate_size	3,072
hidden_dropout_prob	0.1
hidden_act	gelu
max_position_embeddings	512

In the TextCNN model, the dimension of the input vector is 260, the size of four convolution layers in the TextCNN model is 5, 10, 15, and 20, respectively, and the number of convolution layer is half of the average text length (130). In the LSTM layer, the number of units with 260 maximum text length is set to return the output of the last time step. ReLU and AdamW are the activation function and the optimizer, respectively, where W stands for weight decay, and it can adapt to adjust the learning rate of each parameter with good convergence and robustness. All models in the experiment added dropout methods to the FC layer, which dropped 50% of the neurons in order to avoid overfitting. The loss function is the cross entropy which can enable the learning rate of the model controlled by the output error, and the convergence speed is fast which can avoid the problem of low learning rate effectively and keep the model from falling into the local optimal solution. The formula for cross-entropy is shown below:


(3) $$\begin{array}{l} C=-\frac{1}{n}\sum \left[y\ln\;a+\left(1-y\right)\ln\;\left(1-a\right)\right] \end{array}$$

where C is the cost, x is the actual input, a is the actual output, y indicates the expected output, and n is the total number of inputs.

We divided the datasets into the training sets and testing sets by a proportion of 7:3, i.e., the number of which is 348 and 150, respectively. We perform 10 consecutive runs for every classifier and obtain the best performance on the results, and the prediction result is compressed between (0,1) for AD classification.

## 4 Results and discussion

### 4.1 Evaluation metrics

According to the medical clinical diagnosis, the positive result represents the individual with AD and the negative result represents the healthy one. Table 4 shows the relationship between the true class and the predicted class. We can obtain our basic indicators: true-positive (TP), false-positive (FP), false-negative (FN), and true-negative (TN), and these four indicators are presented in Table 4. As the datasets in two classifications are balanced (242 and 256), we choose the accuracy and confusion matrix to evaluate the performance of the LSTM, TextCNN, and LSTM-TextCNN models on the datasets. The accuracy is derived from the confusion matrix:


(4) $$\begin{array}{l} Accuracy=\frac{TP+TN}{TN+FP+FN+TP} \end{array}$$

**Figure 5:**
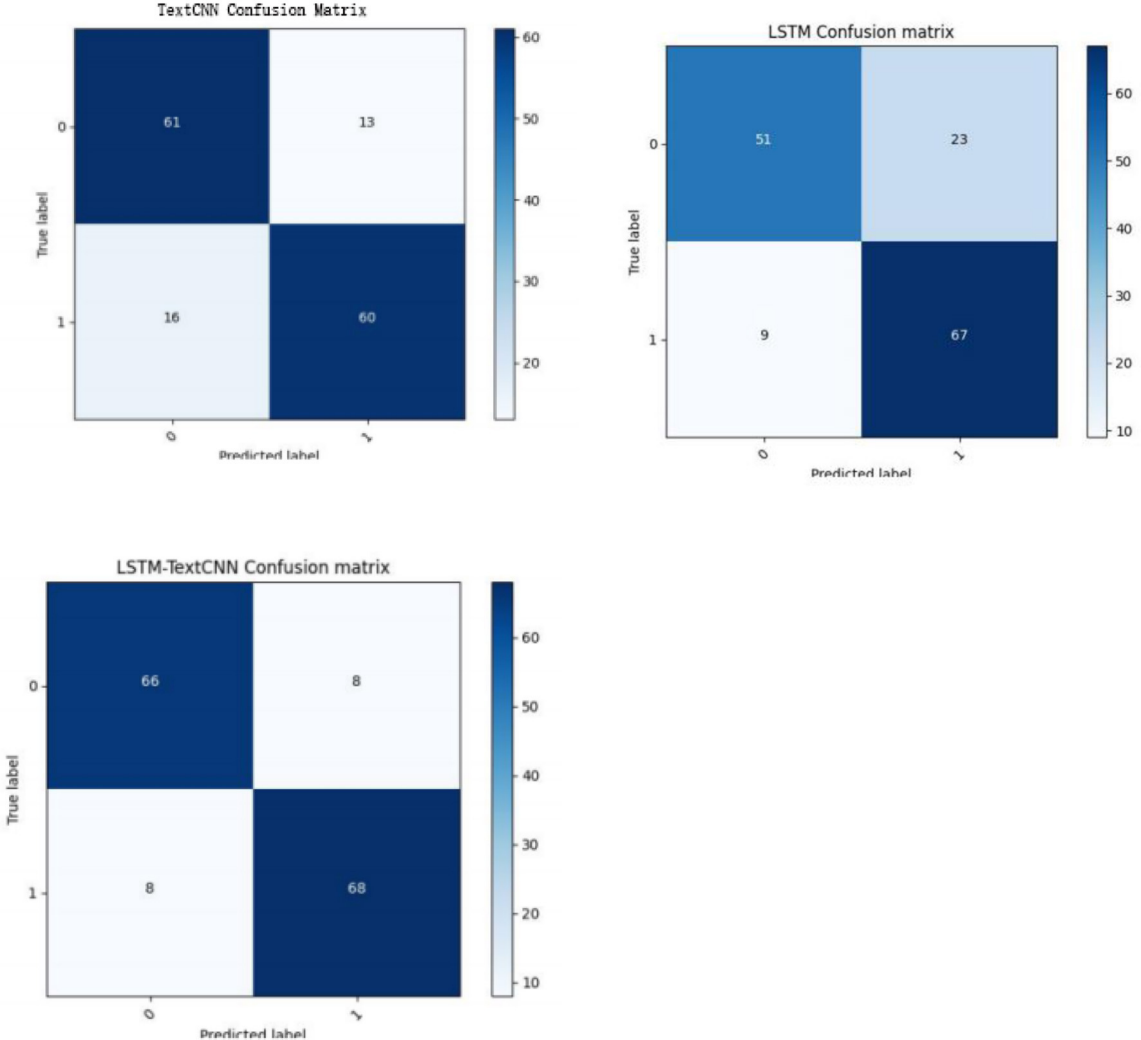
Confusion matrix of the TextCNN, LSTM, and TextCNN-LSTM model.

In the field of machine learning, a confusion matrix is a specific matrix used to visualize the performance of the model. Each row represents the predicted value, and each column is the actual category. In Table 4, TP and TN are the number of samples that are classified correctly by the classifier, so TP+TN is the number of samples classified correctly. Therefore, from Figure 5, we know that of the 150 testing sets, the correct predicted number in the TextCNN, LSTM, and LSTM-TextCNN models is 121(61+60), 118(51+67), and 134(66+68), respectively. According to the formula (6), the accuracy of the three models is 121/150 = 80.7%, 118/150 = 78.7%, and 134/150 = 89.3%, respectively, so the performance of the three models is LSTM-TextCNN > TextCNN > LSTM. The LSTM-TextCNN model has a better performance than the single model mainly because the combined models can integrate different text features extracted from the two models, and the fused features can represent the text more precisely.

**Table 4:** Relationship between the predicted and true class.

**Confusion matrix**	**Actual class**	
**Predicted class**	**Positive**	**Negative**
Positive	True-positive (TP)	False-positive (FP)
Negative	False-negative (FN)	True-negative (TN)

The accuracy of the TextCNN-LSTM model in 10 runs is shown in Table 5, and the reason for 10 runs is that the performance of the model has become stable gradually after 9 runs.

**Table 5:** Accuracy of the TextCNN-LSTM model in 10 runs.

**Runs**	**Accuracy**
1	20%
2	41.3%
3	58%
4	73.3%
5	83.3%
6	85.3%
7	86.7%
8	88%
9	89.3%
10	89.3%

We compare the performance of different machine learning and deep learning models on DementiaBank datasets, and the performance is shown in Table 6; the first eleven rows are the classification matrices with the machine learning method and the remaining rows are matrices with the deep learning method.

**Table 6:** AD vs. CTRL classification scores on DementiaBank datasets.

**Method**	**Embedding**	**Classifier**	**Precision**	**Recall**	**Accuracy**	**AUC**	**F1**
Antonsson et al. (2020)	Semantic features	SVM	-	-	-	0.93	-
Clarke et al. (2021)	286 linguistic features	-	-	-	50–78 for MCI vs. HC, 59–90 for AD vs. HC, and 62–78 for AD+MCI vs. HC	-	-
Haulcy and Glass (2021)	x-vectors and i-vectors features (Snyder et al., 2018)	Random Forests and SVM	-	-	85.4	-	-
Jarrold et al. (2014)	Hand-craft acoustic and linguistic features	Logistic regression	-	-	85.4	-	-
Becker et al. (1994)	35Hand-crafted feature	Logistic regression (LR)	-	-	81.92	-	-
Yancheva and Rudzicz (2016)	12Cluster-based features + LS&A	Random forest	80.00	80.00	80.00	-	80.00
Sirts et al. (2017)	Cluster + PID + SID features	LR	74.4 ± 1.5	72.5 ± 1.2	-	-	72.7 ± 1.2
Hernández-Domínguez et al. (2018)	105Hand-Crafted features	SVM	81.00	81.00	79.00	-	81.00
Li et al. (2019)	185Hand-Craft features	LR	-	-	77	-	-
Fraser et al. (2019)	Info and LM features	SVM	-	-	75	-	77
Fritsch et al. (2019)	n-gram	NNLM+LSTM	-	-	85.6	-	-
Aparna et al. (2021)	-	BERT	-	-	83.33	-	-
Guo et al. (2021)	-	BERT	-	-	82.1	-	-
Amit et al. (2021)	-	FastText	-	-	83.3	-	-
Karlekar et al. (2018)	POS-tagged data	CNN-RNN	-	-	91.1	-	-
Orimaye et al. (2018)	n-grams	D2NN	-	-	88.9	-	-
Pan et al. (2019)	GloVe word embedding sequence	BiLSTM|GRU hierarchical attention	84.02	84.97	-	-	84.43
Yuan et al. (2020)	Encoding of pauses+ERNIE Embedding	ERNIE	-	-	89.6	-	-
Tristan and Saturnino (2021)	Word cooccurrence graphs	Machine learning	-	-	66.7	-	-
Pranav and Veeky (2021)	Linguistic features	Deep learning	-	-	-	88	-
DistilBERT	DistilBERT	-	0.87	0.88	0.8627	-	0.8713
BERT	BERT	-	0.88	0.9149	0.8431	-	0.8776
GPT2	GPT2	-	0.88	0.9149	0.8431	-	0.8776
RoBERTa	RoBERTa	-	0.89	**0.976**	0.8039	-	0.8817
TextCNN	TextCNN	-	0.806	0.822	0.789	-	0.805
LSTM	LSTM	-	0.787	0.744	0.882	-	0.807
Our method	LSTM-TextCNN	Softmax	**0.893**	0.895	**0.895**	-	**0.893**

Bold means the max value in a column.

## 5 Conclusion

This study used the combination of the TextCNN and LSTM model to recognize AD from normal controls (NC), which can combine the advantages of the TextCNN model and LSTM model. First, we pretrained the datasets by the BERT model to obtain the word embedding vector and then used TextCNN to extract local features of different sizes and the LSTM model to obtain the global features. The TextCNN model only obtains the local features of the text, while the LSTM model can obtain the longer features, and the combination of both models can represent the whole text to a certain extent. Therefore, we concatenated the features from the TextCNN layer and LSTM layer to get the feature representation of the entire text, which were put into a Softmax classifier to obtain the classification result. Finally, three models were tested and compared with the evaluation metrics. The experiment results showed that the accuracy was 0.893 which was significantly higher than the LSTM and TextCNN model.

Differences in oral language may supply a tool to differentiate AD for older adults subjects based on the deep learning model, so our study is meaningful in developing a simple but practical, low-cost reliable tool for the early detection of AD or other dementia disease in future based on transcripts of narrative speech. We hope the tool can detect the change of AD gradually with the development of the disease in real time. Based on the above considerations, we believe the use of deep learning method to diagnose AD is an exploring and compelling area for further research study.

## Data availability statement

The original contributions presented in the study are included in the article/supplementary material, further inquiries can be directed to the corresponding author.

## Ethics statement

The manuscript presents research on animals that do not require ethical approval for their study. Written informed consent was obtained from the individual(s) for the publication of any potentially identifiable images or data included in this article.

## Author contributions

NL: Validation, Writing—original draft. LW: Resources, Supervision, Writing—review & editing.

## Footnotes

^1^ https://www.alz.org/alzheimers-dementia/facts-figures
